# Validity and acceptance of self vs conventional sampling for the analysis of human papillomavirus and Pap smear

**DOI:** 10.1038/s41598-023-29255-y

**Published:** 2023-02-16

**Authors:** M. J. Gibert, C. Sánchez-Contador, G. Artigues

**Affiliations:** 1 Coordinació de l’Estratègia de Càncer de les Illes Balears, Directorate General of Public Health, Balearic Health Ministry, 07010 Palma de Mallorca, Illes Balears Spain; 2 Research group on Public Health of Balearic Islands (GISPIB), Health Research Institute of the Balearic Islands (IdISBa), 07010 Palma de Mallorca, Illes Balears Spain; 3 Department of Obstetrics and Gynaecology, Son Espases University Hospital, 07120 Palma de Mallorca, Illes Balears Spain; 4 Faculty of Medicine, University of the Balearic Islands, 07120 Palma de Mallorca, Illes Balears Spain

**Keywords:** Health care, Oncology

## Abstract

The newest high-risk human papillomavirus (HPV) detection techniques were included for cervical cancer primary screening under the Spanish National Health System in 2019. These analyses allow changing population approaches to foster adherence to screening. Therefore, the validity of self versus conventional sampling for HPV and cytology analyses was appraised. Women's preferences concerning samples and devices were also evaluated. This is a diagnostic accuracy cross-sectional study among 120 women recruited from a colposcopy clinic at a general hospital in Illes Balears, Spain. Participants were given written information and asked for a self-sample. One of two sets containing two devices each were handed. One set was transported dry and the second in liquid medium. Next, clinicians collected vaginal samples that were our gold standards. The agreement between both techniques was examined with the Kappa coefficient (κ). Self-sampling evaluation and preferences for different vaginal devices were also surveyed. The agreement between self and conventional samples concerning HPV positivity was very good (κ 0.86 for Mía by XytoTest® and 0.83 for Viba-Brush®) or reasonable (κ 0.73 for Iune and 0.68 for viscose swab). Pap smears from self-samples exhibited moderate agreement (κ 0.41 for Mía® and 0.51 for Viba-Brush® respectively) for negative versus ASC-US and worse results. Most of the participants considered self-sampling as beneficial (110 or 91.7%) and the advantages were, in decreasing order, scheduling, comfort, intimacy and less fear for pain or disturbance. The priority of choice for the devices was Mía® and viscose swab (chosen in first or second place) in opposition to Iune and Viba-Brush® (chosen in third or fourth place). If Viba-Brush® was to collect the best quality samples, 108 women (94.7%) switched their decisions. Our agreement between self and conventional samples was very good or reasonable for HPV, with the best values for devices in a liquid medium, and moderate for cytology. Even so, reflex cytology on self-samples is a valuable tool in promoting adherence. Self-sampling was widely accepted for smooth and thin devices. However, there is no resistance to change to others if a higher quality of the sample is obtained.

## Introduction

In 2019, new techniques for detecting high-risk human papillomavirus (HPV) were included in the portfolio of common services of the Spanish National Health System. Its indication is the primary screening of cervical cancer among women between the ages of 35 and 65, every 5 years^1^. This extension of benefits is based on the European guidelines for quality assurance in cervical screening, 2015^2^, the consensus document of the Spanish Working Group on cervical cancer screening of the National Health System, 2016^3^, and is aligned with the Global strategy to accelerate the elimination of cervical cancer as a public health problem, approved by the World Health Assembly in August 2020^4^.

As cervical cancer predominates among seldom and non-screened women, it is convenient to identify tests of adequate validity to gain and retain them. According to the new portfolio of services^1^, if the HPV test is positive, a Pap smear is indicated and should be performed, if possible, on the same sample, or reflex cytology, to avoid recitations and encourage adherence to the screening^1, 5^.

One resource that may combine clinical validity and acceptance among unscreened and underscreened women could be self-sample or sample taken by the patient, for HPV analysis and cytology. Concerning self-sampling acceptance, in a meta-analysis of 20 research works, 8 found more women preferring self to clinician collection, although they expressed doubts regarding its correct performance and had greater confidence in clinician sampling^6^. In another meta-analysis on over 18,000 women across five continents, participants reported preference for self-sampling over clinician sampling due to its ease and privacy^7^, while in a British population study, 70% of women who actively declined screening expressed interest in self-sampling^8^. In addition, participation in cervical screening achieved by self-sample was 2.1 times higher when compared to women invited to the clinic to undergo a Pap test, a fact that has been documented in a systematic review of ten trials, eight European and two North American, published in 2013^9^.

Different methods have been used to obtain a vaginal smear from patients (e.g., brush, lavage, swab, tampon, and other specially designed devices). Bishop et al.^10^ studied women's preferences for four devices. Acceptance was highest for the simplest ones; that is, for those that most closely resembled a cotton swab and, therefore, consisted of a flexible plastic shaft to direct it and a soft foam matrix to absorb the sample.

Arbyn et al.^11^ conducted a meta-analysis of 81 quality studies and sorted the sensitivity and specificity of the HPV test by device type and storage medium. The relative values for the swab and dry or liquid samples were fully comparable to the clinician samples. The swab materials used were dacron, nylon and cotton. In addition, the women and clinician samples had similar validity if PCR (polymerase chain reaction) assays were used^11^. Prioritizing devices with inert materials, without cotton or wood stalks, seem more prudent, as it is thought that these natural materials may inhibit the PCR^12, 13^.

Concerning cytological studies of self-sampling, it is considered a potentially useful resource, not only for those who are positive for HPV and, therefore, require a reflex cytology but also for those under 35 years of age for whom the primary screening is a Pap smear^1^. We have scarce studies on cytological self-sampling, but we highlight two recent ones. One was performed on 39 residents in Australia who used the sample obtained by Cervibroom, a device commonly used in clinics and deposited it in a liquid medium^14^, and the other on 367 residents in Malaysia^15^ who collected the sample with the Evalyn® Brush device, very similar to the Cervibroom, but much smaller in diameter. In the Australian study^14^, both methods of sampling were compared to the gold standard established in that work, which was colposcopy and an eventual cervical biopsy. Thus, the self-sample exhibited values of sensitivity and specificity for intraepithelial lesion of any degree of 64.7% and 86.4% respectively, while the equivalent figures for conventional sampling were 47.1% and 81.0%. In contrast, in the Malaysian study^15^, self-collection sensitivity of 71.9% and specificity of 86.6% were obtained versus cytology taken by a clinician and showed a concordance, measured with the Kappa coefficient (κ), of 0.57. The above values point to the usefulness of cytological self-sampling to avoid redundant visits and, thus, promoting increased participation in cervical screening.

Variability among the results of self-sampling establishes the need for a pilot study before its implementation in population-based screening programs of a specific region^16, 17^. Published studies on women residents in Spain are scarce. Nevertheless, we have one performed in Mallorca^18^ before 2015 on 120 women undergoing routine screening and 120 diagnosed with a low-grade squamous intraepithelial lesion (LSIL) during the six months prior to their recruitment. Firstly, the women were asked about their preference for sampling, with 72% in favour of doing it on their own. Conventional cytology was used as the gold standard, and the HPV study was performed on the material collected by the clinician and with the Selfcitotest device, obtaining sensitivities of 76% for HPV detection for both, and specificities for the clinician and self-samples of 83% and 85%, respectively. The concordance between both methods, according to κ, was 0.86. Another subsequent study by researchers from the same area^19^, on 196 patients, examined in more detail the validity of the self-sampling with a very similar device, the Iune, and two different brands of PCR, obtaining comparable values to those of the aforementioned study. More recent research from Valencia (Spain) consisted of a telephone survey conducted, in January 2020, on 389 randomly sampled women between the ages of 35 and 65. Almost 87% of these women opted for self-sampling. Younger age, higher level of education and knowledge of screening were the factors that significantly favoured the preference for it^20^.

Based on the above, this research study is considered necessary to investigate in Illes Balears (Spain) the acceptance of self-sampling, the appraisal of the experience, different devices and transport media, the expression of preferences, the handling of the samples by patients, as well as the clinical utility of several kits for HPV and Pap smear analyses.

## Hypothesis and objectives

The *hypotheses* of this study are:

- Cervical-vaginal samples taken by the clinician and by the patients have a similar diagnostic performance concerning the HPV and cytological analyses.
- Women accept vaginal self-sampling as an alternative to clinician sampling.

The *primary objectives* are to establish the diagnostic validity of HPV in vaginal self-samples and the acceptance of its performance by the women. The *secondary objectives* are to scrutinize the validity of self-collection for the Pap smear, to establish women’s preferences on how to get vaginal samples and the types of devices, as well as to foresee the obstacles related to the implementation of self-sampling, such as its management by the patient.

## Methods

### Study design

Diagnostic accuracy cross-sectional study, with prolective data collection to calculate concordance between vaginal self and clinician collection, which was established as the gold standard. The diagnostic accuracy of the vaginal self-sample was analyzed using four devices: two were conveyed dry and two in liquid medium. The kit selection was due to the contrast between the two kinds of transport, given the risk of fluid spillage before, during and after the sampling. Among the devices to be used without medium, a viscose swab was chosen because of its resemblance to cotton swabs and its widespread use in health care centers, and the Iune HPV test cannula for its being designed and manufactured in Spain. With respect to the liquid medium sample, Viba-Brush® was favoured, given its resemblance to Cervibroom, which presumedly increases the chances of obtaining specimens with enough cells, and Mía by Xytotest® for being as thin as the Viba-Brush®, but with a smoother surface and also compatible with the liquid medium. Each participant provided specimens taken by two devices with the same transport medium, thus two subpopulations or groups of patients could be established. All samples were analyzed for HPV, while only those deposited in liquid medium were examined for Pap smear.

A cross-sectional survey was conducted to ask women about self-sampling. The corresponding questionnaire was widely debated among the promoters of the study and piloted for the first days of its performance.

### Sample size

The calculation of the sample size was performed according to the following data:

- HPV self-sampling, with respect to the clinician's sampling and analyzed by PCR-based assays, has a relative sensitivity and specificity of 95% and 92%, respectively, if we consider the most unfavourable scenarios, that is, a vaginal lavage to obtain the sample and the use of cell transport medium^11^.
- Approximately 40% of the women visiting the participating colposcopy clinic have HPV. This estimate was obtained from tests requested in that clinic.
- Patients drop-out was estimated to be close to 0%, since all the health procedures of this research are performed on the same day, consecutively, and the main researcher is directly in charge of the pre-analytical phase.
- Accuracy, or minimum value, of the difference to be detected is 0.1 and the chosen confidence level is 95%.

The estimated number of samples needed to compare each device with the gold standard is 58. Since each participant provides two self-samples, either dry or liquid, two groups of 58 women are needed; that is, a minimum of 116 participants.

### Criteria for participation

Participation was offered to patients scheduled at the colposcopy clinic in the Inca Regional Hospital (Mallorca, Illes Balears) who met the criteria (Table 1).

**Table 1:** Criteria for inclusion and exclusion in the study.

**Inclusion criteria**
Women between 35 and 65 years old
**Exclusion criteria**
Pregnancy and first six weeks of the puerperium
Not having a cervix (prior hysterectomy or trachelectomy)
Vaginal bleeding
Use of medication, creams or vaginal douches in the last 48 h before sample collection
Ignorance of the co-official languages of the Balearic Islands by the participant and, if applicable, by her companion
Illiteracy in the strict or functional sense
Inability to perform self-sampling

### Data and samples collection

Once it was verified that a woman met the criteria for this study, the goal of the research and the sequence of steps was explained to each participant, and everyone was required to read the corresponding fact sheet, and give her written consent.

The allocation of two dry devices and two liquid devices alternated consecutively.

**Figure 1:**
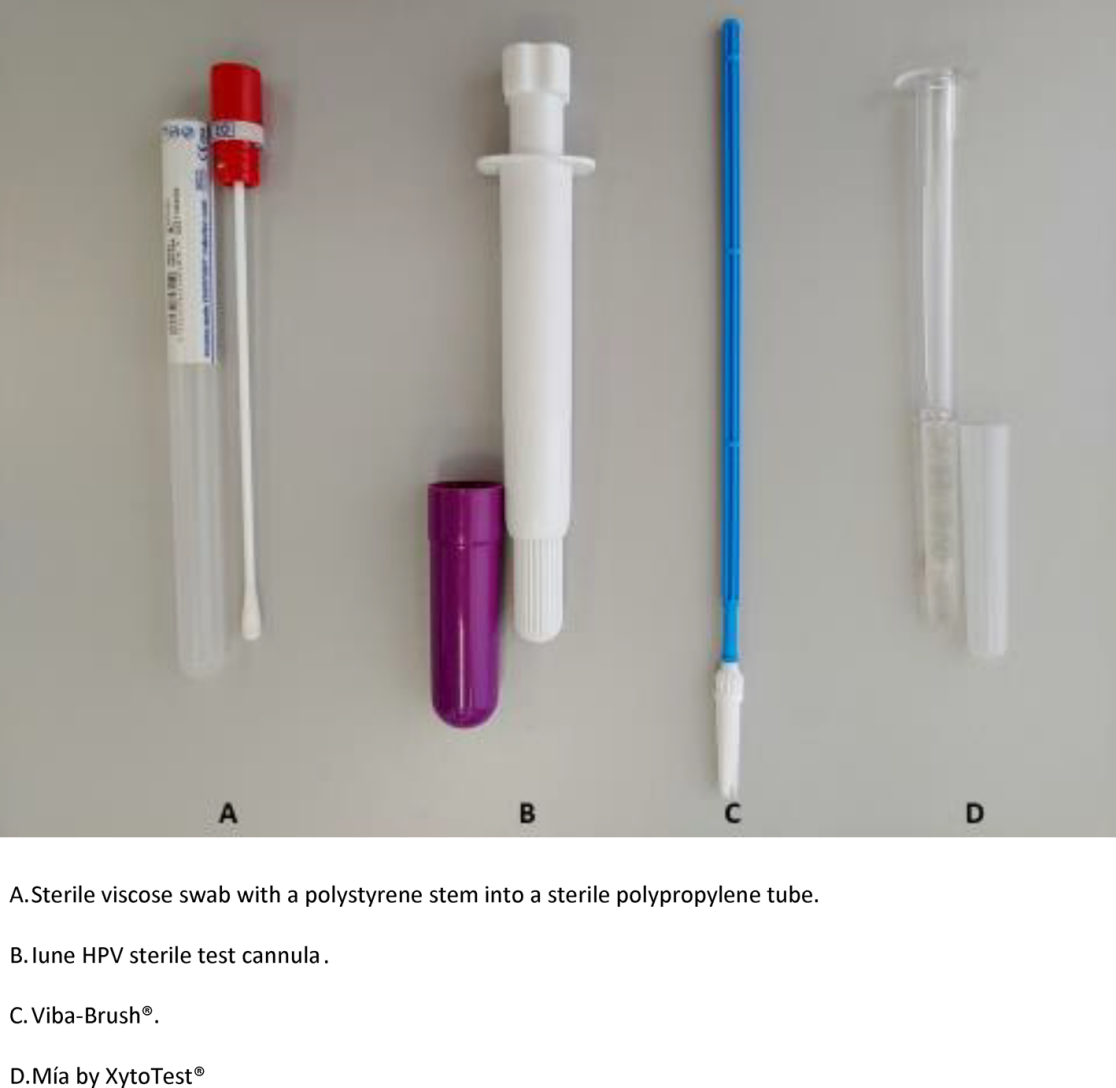
Self-sampling devices included in the study.

The *dry* devices were as follows (Fig. 1):

- A sterile viscose swab with a polystyrene stem into a sterile polypropylene tube and hemispherical bottom, reference number 300252 (Delatalab, Barcelona).
- Iune HPV sterile test cannula (Canda Health Solutions, Mallorca).

The devices in *liquid medium* were as follows (Fig. 1)

(c) Viba-Brush® (Rovers Medical Devices B. V., Oss, The Netherlands) into a vial with 20 mL of PreservCyt® Solution (ThinPrep System, Hologic, MA, USA).
(d) Mía by Xytotest® (Mel-Mont Medical, LLC, Mexico City, Mexico) with a container of 5 mL of PreTect TM® Preservation and Transport Media (PreTect AS, Klokkarstua, Norway).

In order to anonymize the samples for laboratory staff, each was labeled with a number between 1 and 360. This numeral series was the result of a simple random sampling, done at the beginning of the research, and each number was assigned to a delivered device, according to the order of participant recruitment.

The handling of the devices was briefly explained to each woman, to avoid mixing up the samples obtained with one other. Participants were given the instructions for self-sampling in both text and images and told that no additional explanations could be given so that the situation would be as similar as possible to a population-based program of early detection with self-collection.

Samples were obtained in a bathroom near the consulting room. The order of use of the assigned devices was decided by the participant. Later, the investigators surveyed each woman and filled in a questionnaire. The four devices included in this study were shown to be ranked according to preference (two of them had been used and two had not).

The conventional sample was taken during the following hour, as it was assumed that the use of the speculum would be more invasive and, therefore, could reduce the yield of the self-collected sample. It was also presumed that the interference of the self over the conventional sample would be small, being the first less deep and made with a smaller and softer device. The conventional specimen collection was carried out by gently inserting a disposable Cusco's bivalved speculum into the introitus, oriented at a 45º angle. Once inside, it was horizontalized and searched for the cervix, trying not to collide with it. The use of a small amount of water or a water-soluble gel without carbomers was allowed. The device for clinician sampling was the Rovers Cervex-Brush® (Rovers Medical Devices B. V., Oss, The Netherlands), a swab with hydrophobic flexible bristles that allows the simultaneous collection of exo and endocervical cells. The sample, thus obtained, was then deposited into a 20 mL vial of PreservCyt® Solution (ThinPrep System, Hologic, MA, USA). Collaborating gynaecologists also used the endocervical brush regularly on patients with prior negative colposcopies or conized. Before the research started, the participating gynaecologists were instructed to take the sample according to the aforementioned steps.

The samples obtained were conveyed, at room temperature and on the same day, to the Department of Pathology at Son Espases University Hospital. All numbered samples were then transferred to a liquid medium for processing so that the staff in charge of HPV assays did not know the medium in which they had been transported. Analyses of the samples were carried out according to the availability of the laboratory personnel, but always respecting the deadlines set by the manufacturers (six weeks and two weeks at room temperature for cytology and HPV, respectively).

The HPV screening was performed using the Cobas® HPV test (Roche Molecular Systems, CA, USA), which is designed to provide individual results for HPV 16 and HPV 18, along with a pooled result for other 12 high-risk genotypes that are reported as "others".

The cytological analysis was performed with the ThinPrep System (Hologic, MA, USA). The cytology report was written according to the 2014 Bethesda system^21^, so the adequacy of the sample was rated as satisfactory or unsatisfactory for evaluation. The presence or the absence of endocervical cells was also registered.

### Data analyses

The data were entered into an Excel spreadsheet (Microsoft, WA, USA) and analyzed with the statistical resources of its software and other tools available online (Epitools, GraphPad, MedCalc and Social Science Statistics).

The description of the study population was performed through absolute and relative frequencies for the categorical variables, and the median and interquartile range for the quantitative ones. Fisher's exact test for 2 × 2 contingency tables, and the chi-squared test if the tables were of higher order, were used to contrast the proportions of independent groups. On the other hand, if the proportions came from paired samples, the McNemar test was used. The comparison between medians was performed with the Mann–Whitney *U* test. The concordance was scrutinized by the percentage of absolute agreement between the gold standard and the self-samples, and the Kappa coefficient (κ). Negative values were interpreted as no agreement; if they were between 0 and 0.2, as poor agreement; if between 0.21 and 0.40 as fair; if they ranged from 0.41 to 0.60, as moderate; if they were between 0.61 and 0.80, as reasonable; and if they were higher than 0.80, as very good agreement.

P-values were the result of bilateral comparisons, and those below 0.05 were considered statistically significant.

### Study period

Samples were collected between 11/10/20 and 5/18/21, an interval necessary to recruit the number of patients according to the sample size calculation. The statistical analysis was carried out between 8/20/21 and 12/14/21. The interpretation of the results and the writing of the manuscript were undertaken between 9/12/21 and 2/10/22.

### Ethical approval and consent

Biomedical research in Spain is subject to the Organic Law 3/2018, of December 5, on the protection of personal data and the guarantee of digital rights. As a result, the study protocol was evaluated and reported favourably by the Research Ethics Committee of the Balearic Islands. Written informed consent was obtained from all participants. Its text, along with the corresponding fact sheet, is enclosed in this article's supplementary information files. Confidentiality was ensured by anonymizing the participants, once the data were entered into the spreadsheet and its consistency was verified.

## Results

### Final participants

**Figure 2:**
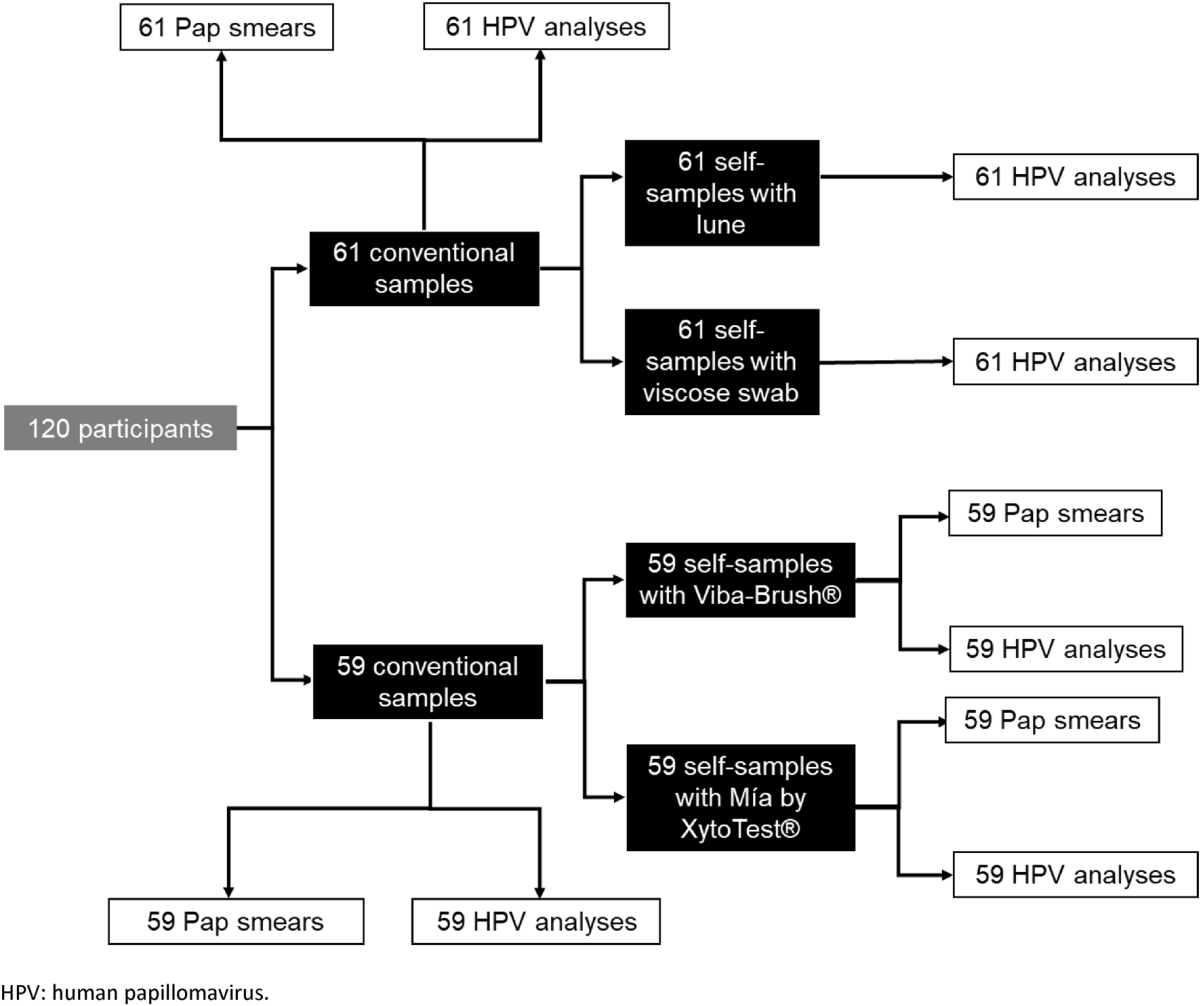
Number of samples and tests distributed among patients.

During the study period, 135 women who met the criteria were located. Ten declined to participate. In addition, five were excluded, as the practitioner didn't consider the sample necessary for clinical reasons. By the end of recruitment, 120 participants had completed all phases, with 61 being assigned to the specimens conveyed dry and 59 to the ones transferred in liquid medium (Fig. 2).

### Socio-demographic characteristics of participants

The median age of the participants was 46 years old, and 62.5% were born in Spain. The predominant level of education was secondary or high school (39.2% of women). As to employment, 47.5% were in a situation of subsidy, mostly due to the COVID-19 pandemic. Table 2 shows the values of these variables in more detail. A comparison is made between the group that was supplied with the dry specimens and the one that received the liquid-based ones. No statistically significant differences were found between the two groups related to socio-demographic traits.

**Table 2:** Socio-demographic characteristics of the participants.

Variables	Total (n = 120)	Dry self-sample (n = 61)	Liquid self-sample (n = 59)	p
**Age median (interquartile range)**	46 (40–51)	46 (40–51)	46 (40–52)	0.6171
**Nationality at birth n (%)**				
Spain	75 (62.5%)	35 (57.4%)	40 (67.8%)	0.4115
Central and South America	26 (21.7%)	17 (27.9%)	9 (15.3%)	
European Union and United Kingdom	9 (7.5%)	4 (6.6%)	5 (8.5%)	
Others	10 (8.3%)	5 (8.2%)	5 (8.5%)	
**Completed educational level n (%)**				
Primary school	36 (30.0%)	13 (21.3%)	23 (39.0%)	0.2120
Secondary school	47 (39.2%)	27 (44.3%)	20 (33.9%)	
Higher education	35 (29.2%)	20 (32.8%)	15 (25.4%)	
No formal schooling	2 (1.7%)	1 (1.6%)	1 (1.7%)	
**Occupation n (%)**				
Employed/self employed	47 (39.2%)	19 (31.1%)	28 (47.5%)	0.2742
Social welfare recipients/retired	57 (47.5%)	32 (52.5%)	25 (42.4%)	
No own income	12 (10.0%)	7 (11.5%)	5 (8.5%)	
Others	4 (3.3%)	3 (4.9%)	1 (1.7%)	

### Evaluation of the pre-analytical phase of cervical-vaginal samples

Only four of the 120 participants (3.3%) did not submit the specimen in due conditions. These cases were evenly distributed between the two groups of devices provided. Two participants delivered partially sealed containers, another discarded the Mía by Xytotest® device after rinsing it in the liquid medium, and the last one handed us the same device dry. However, the aforementioned defects could be corrected before sending the samples to the Pathology Department, except for recovering the discarded device, and it could be verified a posteriori that there was a full coincidence between the samples performed by the woman and the professional.

### Adequacy and results of HPV and cytological analyses

The results of the samples taken both by the collaborating gynaecologists and by the patients are shown in Table 3.

**Table 3:** Adequacy and results of HPV and cytological analyses.

Variables		Conventional samples	Samples in liquid medium		Dry samples	
			Viba-Brush®	Mía by Xytotest®	Iune	Viscose
**HPV test results n (%)**		n = 120	n = 59	n = 59	n = 61	n = 61
Negative		75 (62.5%)	33 (55.9%)	34 (57.6%)	35 (57.4%)	38 (62.3%)
Type 16 ± other types		11 (9.2%)	9 (15.3%)	8 (13.6%)	7 (11.5%)	6 (9.8%)
Type 18 ± other types		4 (3.3%)	2 (3.4%)	2 (3.4%)	3 (4.9%)	3 (4.9%)
Only other types		30 (25.0%)	15 (25.4%)	15 (25.4%)	16 (26.2%)	14 (23.0%)
**Specimen adequacy n (%)**		n = 120	n = 59	n = 59		
Unsatisfactory		24 (20.0%)	1 (1.7%)	1 (1.7%)		
Satisfactory						
Without endocervical component		29 (24.2%)	43 (72.9%)	45 (76.3%)		
With endocervical component		67 (55.8%)	15 (25.4%)	13 (22.0%)		
**Cytological results n (%)**		n = 96	n = 58	n = 58		
Negative		57 (59.4%)	38 (65.5%)	41 (70.7%)		
ASC-US and LSIL		32 (33.3%)	19 (32.8%)	14 (24.1%)		
ASC-H. AIS and HSIL		7 (7.3%)	1 (1.7%)	3 (5.2%)		

*HPV* human papilomavirus, *ASC-US* atypical squamous cells of undetermined significance, *LSIL* low-grade squamous intraepithelial lesion, *ASC-H* atypical squamous cells, cannot exclude a high-grade, *AIS* adenocarcinoma in situ, *HSIL* high-grade squamous intraepithelial lesion.

Twenty per cent of unsatisfactory Pap smears from the samples taken by the clinicians contrasts with the corresponding percentages for specimens collected with Viba-Brush® and Mía by Xytotest®, which are 1.7% for each (only one inadequate cytology per device). So, the 118 pairs of Pap smears from samples taken by the professional and the patient were compared. Then, 24 of 118 self-collected samples (20.7%) became unsatisfactory when taken conventionally, while the only two inadequate results for the self-collection group did not reverse to satisfactory after the gynaecologist's sampling. The p-value for the paired test was significant (p < 0.01).

Concerning endocervical cellularity among all of the satisfactory samples, it was observed that 29 out of 96 conventionally obtained samples (30.2%) did not have enough endocervical cells, while 88 of 116 self-collected samples (75.9%) displayed the same, a difference which was significant (p < 0.0001). Then, 92 pairs of samples were contrasted, and endocervical presence coincided in 18 cases (19.6%). It was found that 4 out of the 22 self-samples with endocervical cells (18.2%) shifted to absence at sampling by the practitioner, while 48 out of the 70 samples without endocervical cells (68.5%) reversed to endocervical presence if obtained by the gynaecologist. The paired contrast was statistically significant (p < 0.01).

Whether the participants had undergone a cone biopsy or not was recorded. Thus, 94 had never been submitted to conization (78.3%), 21 had had one (17.5%), 4 two (3.3%) and 1 three (0.8%). Then, the possible impact of this procedure on the specimen collection quality was scrutinized. Regarding conventional sampling, 18 samples from non operated on women were unsatisfactory (19.1%) versus 6 from operated on ones (23.1%), a difference that was not significant (p = 0.78). Self-sampling also obtained similar satisfactory results, this is, none of the women who had not had this operation obtained an unsatisfactory cytology versus one among the operated on women (3.8%) (p = 0.22). The presence of endocervical cells in the satisfactory samples was also studied. So, 53 of the Pap smears obtained by the clinician in the non-conization group contained them (69.7%) versus 14 of the smears performed in the conization group (70.0%) (p = 1.00). Finally, the proportions were somewhat different among the self-collected specimens, with predominating samples without endocervical cells among 24 of the operated on women (85.7%) compared to the 64 non-operated on (72.7%), although statistical significance was not also reached (p = 0.21).

### Validity of HPV analyses and concordance among them according to the method of sampling

Since all HPV studies were informative, the gold standard was contrasted with the results obtained with all self-sampling devices. Table 4 shows the comparisons among HPV results (negative, positive and different categories of positivity).

**Table 4:** Concordance of human papillomavirus analysis with the gold standard according to each device for self-sampling.

Self-sampling devices	Results of HPV analyses	Conventional sampling					Observed concordance for distinct comparisons (italics)* Sensitivity (95% CI) Specificity (95% CI)	Kappa coefficients (95% CI) Weighted Kappa*
		Negative	Positive	Positive for 16 and/or 18	Positive only for others	Total		
Viscose	Negative	**34 (55.7%)**	5 (8.2%)	1 (1.6%)	4 (6.6%)	39 (63.9%)	*52 (85.3%)* 81.8% (59.7–94.8%) 87.2% (72.6–95.7%)	0.68 (0.49–0.87)
	Positive	4 (6.6%)	**18 (29.5%)**	8 (13.1%)	10 (16.4%)	22 (36.1%)		
	Positive for 16 and/or 18	0 (0.0%)	6 (9.8%)	**6 (9.8%)**	0 (0.0%)	6 (9.8%)	*58 (95.1%)* *50 (82.0%)*	0.77 (0.53–1.00) 0.66 (0.48–0.84) 0.63
	Positive only for others	4 (6.6%)	12 (19.7%)	2 (3.3%)	**10 (16.4%)**	16 (26.2%)		
	Total	38 (62.3%)	23 (37.7%)	9 (14.8%)	14 (23.0%)	61 (100%)		
Iune	Negative	**33 (54.1%)**	6 (9.8%)	1 (1.6%)	5 (8.2%)	39 (63.9%)	*53 (86.9%)* 90.9% (70.8–98.9%) 84.6% (69.5–94.1%)	0.73 (0.55–0.90)
	Positive	2 (3.3%)	**20 (32.8%)**	9 (14.8%)	11 (18.0%)	22 (36.1%)		
	Positive for 16 and/or 18	0 (0.0%)	6 (9.8%)	**6 (9.8%)**	0 (0.0%)	6 (9.8%)	*57 (93.4%)* *50 (82.0%)*	0.72 (0.46–0.97) 0.67 (0.50–0.84) 0.66
	Positive only for others	2 (3.3%)	14 (23.0%)	3 (4.9%)	**11 (18.0%)**	16 (26.2%)		
	Total	35 (57.4%)	26 (42.6%)	10 (16.4%)	16 (26.2%)	61 (100%)		
Viba-Brush®	Negative	**32 (54.2%)**	4 (6.8%)	0 (0.0%)	4 (6.8%)	36 (61.0%)	*54 (91.5%)* 95.7% (78.1–99.9%) 88.9% (73.9–96.9%)	0.83 (0.68–0.97)
	Positive	1 (1.7%)	**22 (37.3%)**	11 (18.6%)	11 (18.6%)	23 (39.0%)		
	Positive for 16 and/or 18	0 (0.0%)	9 (15.3%)	**9 (15.3%)**	0 (0.0%)	9 (15.3%)	*57 (96.6%)* *52 (88.1%)*	0.88 (0.72–1.00) 0.79 (0.65–0.94) 0.76
	Positive only for others	1 (1.7%)	13 (22.0%)	2 (3.4%)	**11 (18.6%)**	14 (23.7%)		
	Total	33 (55.9%)	26 (44.1%)	11 (18.6%)	15 (25.4%)	59 (100%)		
Mía by XytoTest®	Negative	**33 (55.9%)**	3 (5.1%)	0 (0.0%)	3 (5.1%)	36 (61.0%)	*55 (93.2%)* 95.7% (78.1–99.9%) 91.7% (77.5–98.3%)	0.86 (0.73–0.99)
	Positive	1 (1.7%)	**22 (37.3%)**	10 (16.9%)	12 (20.3%)	23 (39.0%)		
	Positive for 16 and/or 18	0 (0.0%)	9 (15.3%)	**9 (15.3%)**	0 (0.0%)	9 (15.3%)	*58 (98.3%)* *54 (91.5%)*	0.94 (0.82–1.00) 0.85 (0.72–0.98) 0.82
	Positive only for others	1 (1.7%)	13 (22.0%)	1 (1.7%)	**12 (20.3%)**	14 (23.7%)		
	Total	34 (57.6%)	25 (42.4%)	10 (16.9%)	15 (25.4%)	59 (100%)		

Coincidental results between samples are in bold.

*HPV* high-risk human papillomavirus, *95% IC* 95% confidence interval.

*The values corresponding to the comparison between positive and negative HPV tests are shown, later those HPV positive for types 16 and/or 18 versus the rest of the results and, finally, among the three possible categories (negative, positive for types 16 and/or 18, positive only for others).

The validity measures and κ coefficients for the various devices ordered from highest to lowest are Mía by XytoTest®, Viba-Brush®, Iune and viscose. For the first two devices, the sensitivity is similar, 95.7%, although the specificity is slightly higher for Mía by XytoTest®, 91.7% versus 88.9%. The concordance is very good for both (0.86 and 0.83 respectively). On the other hand, between the last two, the decrease in sensitivity is more marked; 90.9% for Iune compared to 81.8% for viscose swab, although the specificity is better for the viscose device, that is, 87.2% compared to 84.6%. However, the concordance is reasonable for both (0.73 and 0.68 respectively). It is interesting to note that the contrast between the positive assays for HPV types 16 and/or 18 and the rest improves the values for all the devices but Iune, reaching a maximum value of 0.94 for Mía by XytoTest®.

### Concordance among liquid cytology results according to sampling method

A statistical analysis was performed on the informative samples, whose figures and results of the κ coefficients are depicted in Table 5. The smear interpretation was grouped into three categories, that is, negative for intraepithelial lesion or malignancy; ASC-US (atypical squamous cells of undetermined significance) and LSIL (low-grade squamous intraepithelial lesion); ASC-H (atypical squamous cells, cannot exclude high-grade intraepithelial lesion), atypical glandular cells, and HSIL (high-grade squamous intraepithelial lesion). As it may be seen, the values are higher for Viba-Brush® than for Mía by Xytotest® (weighted κ of 0.43 and 0.30, respectively), and correspond to moderate and fair agreements respectively. The contrast between negative results versus the group including ASC-US and dysplasia showed slightly higher values, that is, κ of 0.51 for Viba-Brush® and 0.41 for Mía by Xytotest®. However, the specificity values were identical for both devices, i.e., 84.6%, but the sensitivity figures were slightly higher for Viba-Brush® than for Mía by Xytotest®, i.e., 65.0% versus 55.0%.

**Table 5:** Concordance of cytological analysis with the gold standard according to each device for self-sampling.

Self-sampling devices	Results of the cytological analysis	Conventional sampling					Observed concordance for distinct comparison (italics)* Sensitivity (95% CI) Specificity (95% CI)	Kappa coeficient (95%CI) Weighted Kappa*
		Negative	Positive	ASC-US or LSIL	ASC-H, AGC or HSIL	Total		
Viba-Brush®	Negative	**22 (47.8%)**	7 (15.2%)	7 (15.2%)	0 (0.0%)	29 (63.0%)	*35 (76.1%)* 65.0% (40.8%-84.6%) 84.6% (65.1%-95.6%)	0.51 (0.25–0.76)
	Positive	4 (8.7%)	**13 (28.3%)**	11 (23.9%)	2 (4.3%)	17 (37.0%)		
	ASCUS or LSIL	4 (8.7%)	13 (28.3%)	**11 (23.9%)**	2 (4.3%)	17 (37.0%)	*33 (71.7%)*	0.43 (0.19–0.68) 0.46
	ASC-H, AGC or HSIL	0 (0.0%)	0 (0.0%)	0 (0.0%)	**0 (0.0%)**	0 (0.0%)		
	Total	26 (56.5%)	20 (43.5%)	18 (39.1%)	2 (4.4%)	46 (100%)		
Mía by XytoTest®	Negative	**22 (47.8%)**	9 (19.6%)	9 (19.6%)	0 (0.0%)	31 (67.4%)	*33 (71.7%)* 55.0% (31.5%-76.9%) 84.6% (65.1%-95.6%)	0.41 (0.15–0.67)
	Positive	4 (8.7%)	**11 (23.9%)**	9 (19.6%)	2 (4.3%)	15 (32.6%)		
	ASCUS or LSIL	4 (8.7%)	10 (21.7%)	**8 (17.4%)**	2 (4.3%)	14 (30.4%)	*30 (65.2%)*	0.30 (0.06–0.55) 0.36
	ASC-H, AGC or HSIL	0 (0.0%)	1 (2.2%)	1 (2.2%)	**0 (0.0%)**	1 (2.2%)		
	Total	26 (56.5%)	20 (43.5%)	18 (39.1%)	2 (4.3%)	46 (100%)		

Coincidental results between samples are in bold.

*ASC-US* atypical squamous cells of undetermined significance, *LSIL* low-grade squamous intraepithelial lesion, *ASC-H* atypical squamous cells, cannot exclude high-grade intraepithelial lesion, *AGC* atypical glandular cells, *HSIL* high-grade squamous intraepithelial lesion, *95% IC* 95% confidence interval.

*Firstly, the values corresponding to the comparison between positive and negative Pap smears are shown, later those among three categories (negative; ASC-US and LSIL; ASC-H, AGC and HSIL).

**Figure 3:**
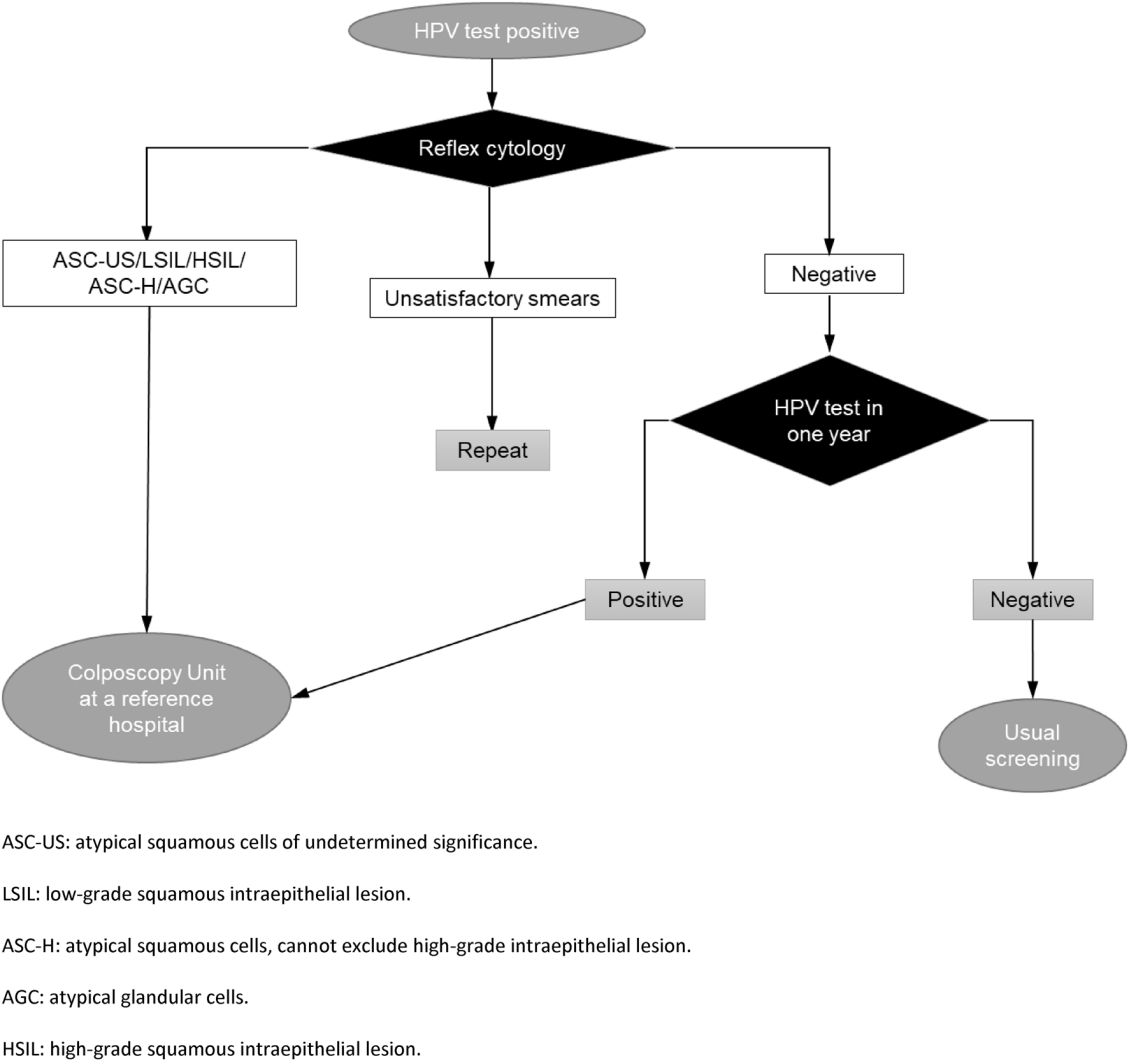
Management algorithm for positive HPV assays in women aged 35–65 years.

The health repercussions of using the patient-collected samples instead of the conventional ones were also explored. For this, the algorithm established in the corresponding Spanish rules was applied^1^ (Fig. 3).

*Negative repercussions of self-sampling* were defined as one or more indications for referral to colposcopy according to the conventional sampling that did not correspond to a similar indication after self-sampling, that is:

- A negative HPV result from self-sampling and a positive result for 16 and/or 18 HPV types from conventional sampling.
- A conventional cytological result of HSIL, ASC-H or AGC and lack of indication for referral in the same year according to the paired self-collected sample.

It was observed that there were no negative repercussions for the devices, but for the viscose one, on account of a single self-taken specimen that was negative for HPV while the corresponding conventional one exhibited the results of HPV positive for other types and a cytological HSIL.

### Self-sampling women’s reporting

All 120 participants were asked if they knew that self-sampling could be used for the early detection of cervical cancer, and the answer was unanimously negative. We continued by asking the women to allocate the self-collection in three categories; thus, for 110, it was an advantage (91.7%), for 7, neither advantage nor disadvantage (5.8%), and for 3, an inconvenience (2.5%). The last three justified their answer by wanting the professional to take the sample, two of them feared they would not do well, and the third thought she might get hurt.

The 110 participants who rated the self-collection as advantageous were asked to select one or more reasons for their rating. The benefits invoked, from highest to lowest frequency, were: schedule or not depending on the timetable of the health centre nor the menses (66/110; 60.0%); comfort or being able to choose the time and place of the sampling (also 66/110; 60.0%); intimacy or preference not to expose the genitals to the health staff (42/110; 38.2%); fear of suffering pain or discomfort while being sampled by the clinician (24/110; 21.8%); and others (6/110; 5.5%).

In addition, they were inquired if they were or not familiar with the use of menstrual care devices (tampons and/or menstrual cups), with 90 women responding in the affirmative (75%) and 30 in the negative (25%). Of the 90 users of period products, 85 rated self-collection as an advantage (94.4%), while among the 30 non-users, 25 also rated it favourably (83.3%). The difference between the two proportions did not reach statistical significance (p = 0.1866).

Once the self-collection was over, they were requested if they had noticed any pain or discomfort. Ninety-one participants answered no (75.8%), and 29 said yes (24.2%). With respect to age influencing the rating, it was observed that the median age for the group considering the sampling annoying and/or painful was 44 years old (39–52) and 48 years old (42–51) for those who noticed no discomfort or pain, differences which were not significant (p = 0.1738). The stated causes of pain and/or discomfort were vaginal dryness associated with menopause, breastfeeding, contraception or just passed menstrual bleeding; the discomfort of the hospital toilet (space, cleanliness, difficulty hanging clothes or bag); comorbidities such as osteoarthritis of the hands or fibromyalgia; ignorance of vaginal anatomy; or preferring to take the sample at home and being able to change body position during sampling. We asked if they had had difficulty performing the self-collection, so 116 had no difficulty performing it (96.7%), while the remaining 4 (3.3%) did. Of the last four, two had been assigned to dry and two to liquid devices. They were between 46 and 63 years old and reported pain and/or discomfort. Three were unfamiliar with the use of vaginal period products, two reported common discomfort during gynaecological examinations and one reported difficulty in interpreting the instructions. Still, all four considered self-collection as an advantage.

Finally, they were asked if they would request self-sampling on another occasion, obtaining an affirmative answer in 102 women (85.0%), a negative in 16 (13.3%) and an indeterminate one for the remaining 2 (1.7%). Of the 16 who said no, 14 argued they relied more on sampling by the practitioner. One commented that self-collection was a pretext to depersonalize care and avoid contact with patients and the second alluded to the side benefits of face-to-face visits (visual inspection, possibility of getting informed about gynaecological issues). Other arisen aspects were the possible obstacles linked to pelvic floor disorders, the alleged duplication of care if a woman frequented the gynaecology offices for reasons other than screening, the concern of becoming infected while self-sampling if the woman is immunocompromised and laziness to read the instructions. The answer was segmented according to age and educational level. Thus, 53 women (93.0%) under 46 would resort to self-collection again compared to 49 (77.8%) of the oldest, a difference that was statistically significant (p = 0.0226). Concerning the level of education, 30 of 38 women (78.9%) who had primary school or lower would ask for self-sampling again versus 72 of 82 (87.8%) with higher education. However, the disparity between the two proportions was not significant (p = 0.2715).

### Device preferences

The order of choice of the device was recorded for 119 participants, 60 of whom used the viscose and Iune devices, and 59 the Viba-Brush® and the Mía by Xytotest®. Table 6 specifies preferences, absolute numbers, and percentages in-depth.

**Table 6:** Devices for self-samples ordered from highest to lowest preference of participants.

Device	Group viscose and Iune (n = 60)	Group Viba-Brush® and Mía by Xytotest® (n = 59)	Totals
**1st preference**			
Viscose	**38 (63.3%)**	4 (6.8%)	42 (35.3%)
Iune	17 (28.3%)	7 (11.9%)	24 (20.2%)
Mía by Xytotest®	2 (3.3%)	**45 (76.3%)**	**47 (39.5%)**
Viba-Brush®	3 (5.0%)	3 (5.1%)	6 (5.0%)
Total	60 (100%)	59 (100%)	119 (100%)
**2nd preference**			
Viscose	12 (20.0%)	**21 (35.6%)**	**33 (27.7%)**
Iune	**20 (33.3%)**	12 (20.3%)	**32 (26.9%)**
Mía by Xytotest®	18 (30.0%)	11 (18.6%)	29 (24.4%)
Viba-Brush®	10 (16.7%)	15 (25.4%)	25 (21.0%)
Total	60 (100%)	59 (100%)	119 (100%)
**3rd preference**			
Viscose	6 (10.0%)	18 (30.5%)	24 (20.2%)
Iune	12 (20.0%)	**24 (40.7%)**	**36 (30.3%)**
Mía by Xytotest®	**27 (45.0%)**	3 (5.1%)	30 (25.2%)
Viba-Brush®	15 (25.0%)	14 (23.7%)	29 (24.4%)
Total	60 (100%)	59 (100%)	119 (100%)
**4th preference**			
Viscose	4 (6.7%)	16 (27.1%)	20 (16.8%)
Iune	11 (18.3%)	16 (27.1%)	27 (22.7%)
Mía by Xytotest®	13 (21.7%)	0 (0.0%)	13 (10.9%)
Viba-Brush®	**32 (53.3%)**	**27 (45.8%)**	**59 (49.6%)**
Total	60 (100%)	59 (100%)	119 (100%)

The highest figures have been highlighted in bold.

Due to the similarity between Viba-Brush® and Cervex-Brush®, participants were told that Viba-Brush® would probably get the same quality of conventionally collected specimens. As a result, they were asked if, in the case that they had not prioritized Viba-Brush®, they would swap to it to get a better cervicovaginal specimen. Of the 114 women who did not first choose Viba-Brush®, 108 (94.7%) accepted the change, and the remaining 6 (5.3%) did not.

## Discussion

The HPV analyses showed a reasonable or very good agreement with the gold standard for all the devices scrutinized, with the best result for Mía by Xytotest® and the worst for the viscose swab. Our values are in line with the meta-analysis of Petignat et al.^22^ who calculated a κ statistic of 0.66 for the self-samples. A much more recent Japanese study^23^, used the same HPV test and material for conventional sampling as in this study, but the self-collection device, Evalyn® Brush, which is quite similar to Viba-Brush®. A κ value of 0.76 was obtained in that study while, in the present work, it was 0.83 for Viba-Brush®.

In contrast, the values of cytological concordance were lower than HPV, a fact already documented in previous research works on this subject^24, 25^. Thus, the comparison between negative smear results and ASC-US or worse returned κ values of 0.51 and 0.41, which are slightly lower than 0.65 from the study by Pengsaa et al.^26^ or 0.57 of Othman et al.^15^. The curtailed validity of cytology versus HPV, however, should not lead us to ignore the possibilities of reflex cytology in self-sampling. The sensitivity and the specificity of our self-collected smears show very similar figures to those of other works with the same gold standard^15, 27^, allowing us to avoid visits only for Pap smear collection, whenever the self-sample resulted in a smear with non-reactive atypia or dysplasia^24, 25^.

During our inquiries about the validity of the scrutinized tests, we observed a much higher ratio of unsatisfactory smears among specimens taken by the gynaecologists than by the women. This fact contrasts with the informative results of HPV tests for every kind of sampling. This disparity draws even more attention if we consider that cytological reports were performed by an experienced professional team, whose historical ratio of inadequate samples is around 1%^28^. Moreover, the fraction of satisfactory smears from self-collection switching to unsatisfactory after conventional sampling and the lacking reverse phenomenon allows hypothesizing the detrimental effect of immediate prior self-collection on practitioner sampling adequacy. This incident is attributed to cell depletion (three specimens collected from every patient in an hour) and/or to the diligence of women related to the sampling depth. Conversely, this fact has no counterpart in the HPV test, a method with fewer requirements.

On the contrary, the satisfactory samples obtained by the gynaecologists had higher endocervical cellularity than those collected by the patients, a totally expected difference due to the direct contact of the Cervex-Brush® with the endocervix and the use of the endocervical brush in certain cases. In the work by Singla et al.^14^, endocervical cells were retrieved in 15% of the self-samples, which points to a certain potential of self-collection to gather cells from the upper end of the lower genital tract. The cone biopsies did not influence the adequacy of the sample, regardless of the method of collection, nor, in a relevant way, in the endocervical cells found in the self-samples. It has traditionally been thought that the absence of endocervical cells could compromise the diagnosis of incipient cervical adenocarcinomas and its precursor lesions. However, research does not support this assertion^29–33^. It is also postulated that HPV screening in cervical secretions has the potential to increase the diagnosis of both in situ and invasive glandular lesions^34^, which would further counteract, if possible, the importance of cytological endocervical presence.

Among the participants of this study, self-sampling for detecting precursor lesions of cervical cancer seems completely unknown, as all of them were unaware of this option. However, most women considered it an advantage and stated they would request it next time, with very similar figures to a recent survey in the Comunidad of Valencia (Spain)^20^. As in this last study, the younger age of the participants favoured the self-collection choice, a fact that supports its growing acceptance over time.

The benefits mentioned among those who considered the patient-collected sampling an advantage are linked to the schedule, comfort, intimacy and less hassle. However, among those who were reluctant to use it in the future, the main argument was greater confidence in sampling by a professional. Similar reasons were given in other settings where this sort of sample has been appraised^7, 10, 35, 36^.

As for the devices, the order of preference was, from more to less: Mía by Xytotest®, viscose swab, Iune and Viba-Brush®. This is interpreted as women preferring thinner, smoother-surfaced devices, regardless of their intended use, general or specific for vaginal sampling, age, or perimenopausal condition. The study by Bishop et al.^10^ shows a greater preference for the Catch-All™ Swab device, very similar to the viscose swab, and the Qvintip®, both thinner and smoother than the other devices considered. These inclinations are consistent with around one-quarter of participants reporting pain or discomfort during the collection, symptoms unrelated to the age of the patients and attributed by themselves to vaginal dryness that may occur at different stages of life (menopause, breastfeeding, contraceptive use or the postmenstrual stage). However, women could sacrifice their preference to obtain a better-quality sample.

In short, HPV screening on self-collected specimens and eventual sequential Pap smear, if HPV is positive, will allow choosing which women will need a colposcopy with the same effectiveness as the face-to-face visits. In addition, this work endorses the feasibility of self-sampling in our area, its potential to increase participation of women in cervical screening and optimize the resources of in-person consultation.

### Limitations

It is likely that the use of the last specimen of three consecutive ones for a Pap smear was responsible for the unexpectedly high ratio of unsatisfactory samples. We think that these figures would have improved if the sampling by the practitioner had been postponed for a few weeks, although the study would have lasted longer due to patients’ drop-out. Likewise, as the n was calculated to assess the validity of the HPV test in the self-collected samples, it was insufficient to display the full potential of its cytological version.

In real screening, in which the algorithm in Fig. 3 must be met, cytologists and cytotechnicians will not only be aware of the HPV carrier status but will probably also know the viral type. This will increase the sensitivity of Pap smears, as subtle cellular changes will be interpreted differently.

Finally, we should bear in mind that our participants were recruited from a colposcopy clinic, therefore their attitude towards screening and self-sampling may be more favourable than in the rest of the population.

## Conclusion

The concordance between the self and clinician samplings was reasonable or very good for HPV, achieving the best values for the samples kept in a liquid medium. As to cytology, concordances were moderate and fair. However, reflex cytology has the utility of promoting adherence to screening by reducing avoidable appointments, if the cytological self-sample is positive.

Cervical-vaginal self-collection is remarkably accepted among our participants who, although they prefer slimmer devices with smoother surfaces, do not hesitate to replace them with less appreciated ones if this results in a higher quality specimen.

### Consent for publication

All participants were provided with a fact sheet about this research, accepted their participation, approved of the use of data acquired, and signed an informed consent in the co-official languages in the Balearic Islands (Spain).

## Data Availability

All data generated or analysed during this study are available upon reasonable request.
