# Author Correction: Validity and acceptance of self vs conventional sampling for the analysis of human papillomavirus and Pap smear

**DOI:** 10.1038/s41598-023-37880-w

**Published:** 2023-07-05

**Authors:** M. J. Gibert, C. Sánchez-Contador, G. Artigues

**Affiliations:** 1 Coordinació de l’Estratègia de Càncer de les Illes Balears, Directorate General of Public Health, Balearic Health Ministry, 07010 Palma de Mallorca, Illes Balears Spain; 2 Research Group on Public Health of Balearic Islands (GISPIB), Health Research Institute of the Balearic Islands (IdISBa), 07010 Palma de Mallorca, Illes Balears Spain; 3 Department of Obstetrics and Gynaecology, Son Espases University Hospital, 07120 Palma de Mallorca, Illes Balears Spain; 4 Faculty of Medicine, University of the Balearic Islands, 07120 Palma de Mallorca, Illes Balears Spain

Correction to: *Scientific Reports* 10.1038/s41598-023-29255-y, published online 16 February 2023

The original version of this Article contained an error in the Funding section.

“This study was carried out by staff employed by the General Directorate of Public Health and the Health Service of the Balearic Islands. No additional sources of funding have been required.”

now reads:

“This study was carried out by staff employed by the Health Service of the Balearic Islands and the General Directorate of Public Health, the organization which supported the costs of the open access publication."

The original Article has been corrected.

